# Third annual transplant AI symposium: from organ matching to digital twins

**DOI:** 10.3389/frtra.2026.1871888

**Published:** 2026-06-26

**Authors:** Devina Ramesh, Praveen Manickavel, Aman Sidhu, Walter K. Kremers, Girish Mour, Robert C. Huebert, Joseph Ahn, Mamatha Bhat

**Affiliations:** 1 Transplant AI Initiative, Ajmera Transplant Program, University Health Network, Toronto, ON, Canada; 2 School of Medicine, Queen’s University, Kingston, ON, Canada; 3 Schulich School of Medicine & Dentistry, Western University, London, ON, Canada; 4 Department of Medicine, University of Toronto, Toronto, ON, Canada; 5 Medical Director of Lung Transplant Program, University of Toronto, Toronto, ON, Canada; 6 Lead Statistician at Mayo Clinic’s Department of Quantitative Health Sciences and Associate Professor of Biostatistics Rochester, MN, United States; 7 Department of Internal Medicine, Mayo Clinic College of Medicine and Science, Rochester, AZ, United States; 8 William J. von Liebig Center for Transplantation and Clinical Regeneration, Mayo Clinic College of Medicine and Science, Rochester, MN, United States; 9 Toronto General Hospital Research Institute, University Health Network, Toronto, ON, Canada; 10 Division of Gastroenterology and Hepatology, University Health Network, Toronto, ON, Canada

**Keywords:** AI, TAI symposium, transplant, transplant artificial intelligence, transplant medicine

## Abstract

The Ajmera Transplant Center and Mayo Clinic hosted the third annual Transplant Artificial Intelligence (AI) Symposium in Toronto, Canada, bringing together expert clinicians, researchers, scientists, and trainees to discuss the current role of AI in transplant medicine. This paper summarizes the third annual Transplant AI Symposium proceedings and talks. Presentations covered a wide range of topics across the transplant continuum, highlighting numerous benefits of AI in transplantation such as organ matching, human-AI collaboration, and survival/risk prediction. Artificial intelligence is most useful when linked to specific clinical problems, especially those involving multimodal or longitudinal data. However, speakers also emphasized ongoing limitations in data quality, generalizability, workflow integration, and fairness. Multiple presentations highlighted the importance of clinician oversight. Overall, the symposium highlighted that the future of transplant AI will depend on careful validation, clinically meaningful implementation, and attention to patient outcomes (Figure 1).

## 1 Introduction

The Ajmera Transplant Centre and Mayo Clinic hosted the third annual Transplant AI Symposium in Toronto, Canada, continuing the momentum from previous symposia. Bringing together clinicians, researchers, data scientists, and trainees to discuss the growing role of AI in transplant care, these symposia emphasize discovery, collaboration, and real-world implementation of AI. The decisions made in the field of transplantation medicine hinge on high-dimensional clinical information, spanning donor factors, recipient characteristics, longitudinal data, imaging, and allocation decisions. Transplantation is a well-suited field for the meaningful use of AI as an added member of the team, not as a replacement for clinical judgement.

**Figure 1:**
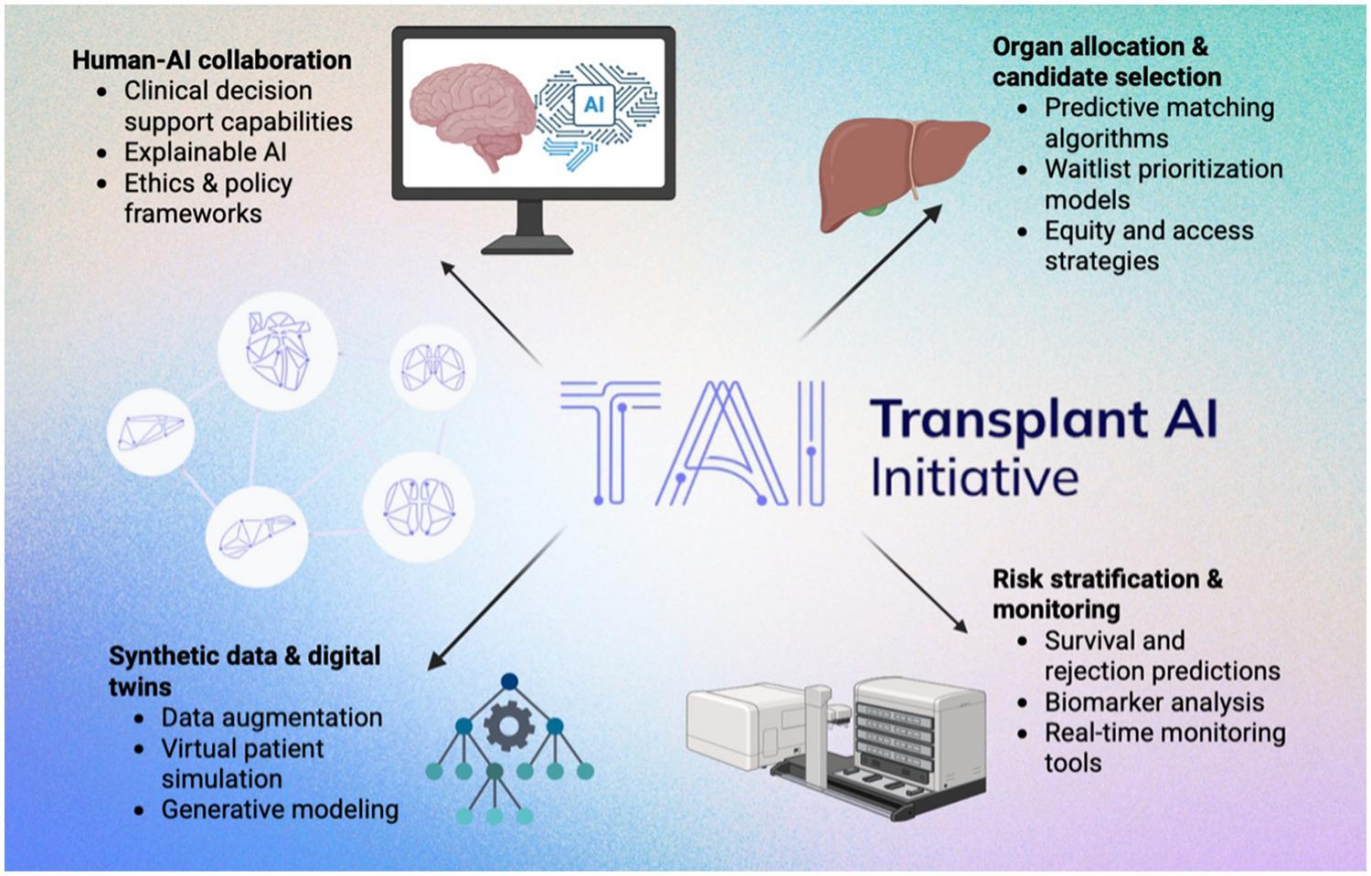
Transplant AI initiative 2025 symposium graphical abstract.

The most promising applications of AI were not isolated prediction tools, but models embedded within real clinical situations. Our Transplant AI Symposium addressed organ matching, post-transplant viral risk stratification, liver transplant candidacy, immunosuppression management, synthetic data/digital twins, the statistical foundations of machine learning (ML) models, human-AI collaboration, non-invasive biomarkers of graft injury, long-term cardiovascular risk post-liver transplant, and prediction of post-transplant lung function. Taken together, these talks addressed where AI fits within medicine, how they should be evaluated, and which problems can be solved in a clinically meaningful way (Table 1).

**Table 1:** Comprehensive overview of the speakers and presentations at the 2025 transplant AI symposium.

**Speaker**	**Model name**	**Domain**	**Specific functionality**	**Application in transplant medicine**	**Performance metric**	**Validation/Deployment**	**Limitations/Methodological considerations**
Dr. Tuomas Sandholm	Branch-and-Price	Kidney exchange optimization	Exact optimization of cycle- and chain-capped exchange graphs	Large-scale kidney donor–recipient matching	Exact optimal batch matching	Live in kidney exchange since 2010; core of US kidney exchange programs	Still in simulation stage of study, not yet peer-reviewed. Limited generalizability at this time.
	Probabilistic expected-weight matching	Kidney exchange/donor-chain optimization	Incorporates edge success probabilities, failure risk, and future potential	Failure-aware donor-chain planning	∼2-fold increase in successful matches in simulation	Simulation stage; not fielded yet	Still in simulation stage of study. Needs further evaluation.
Dr. Aman Sidhu	Monte Carlo lung waitlist simulator	Lung transplant/CMV/allocation	Simulates candidate arrival, donor arrival, mortality, priority change, and optional CMV matching	Tests donor–recipient CMV matching policies	C-index 0.86 for mortality; 0.77 for status change; simulated transplant rate 92.1% vs. observed 92.2%	Retrospective simulation framework; not yet deployed	Retrospective simulation framework. Still requires prospective evaluation.
Dr. Mamatha Bhat	Multi-agent AI transplant selection committee	Liver transplant candidacy	Role-specific AI agents emulate multidisciplinary committee review and consensus decision-making	Improves objectivity in transplant candidacy assessment	92% accuracy for 1-year survival benefit; 91% in UHN silent trial	Reviewed >8,000 SRTR cases; silent trials underway at UHN and multiple international centres	Early trial stage. Needs further evaluation.
	DynaMELD	Liver allocation/waitlist prioritization	Dynamic end-stage liver disease model using rates of change and broader clinical variables	Predicts 90-day waitlist death or dropout more dynamically than static MELD	90-day concordance index 0.825–0.827	Trained on >53,000 US waitlisted patients	Retrospective waitlist model trained on historical data. Still requires prospective evaluation.
	Weighted Long Short-Term Memory Network	Post-transplant graft fibrosis	Dynamic diagnosis of stage 2 fibrosis from longitudinal post-transplant data	Detects graft fibrosis in liver transplant recipients	AUC 0.798	Internal model development	Not validated clinically. External validation still required.
	GraftIQ	Graft injury classification	Multiclass ANN using longitudinal clinical and laboratory features; later refined with clinical rules	Differentiates causes of elevated liver enzymes and graft injury	AUC 0.866 across 6 classes. Hybrid GraftIQ, with clinician input, achieved AUC of 0.902	Externally validated at Mayo, NUHS Singapore, and Hanover	Proof-of-concept model. Needs further evaluation.
Dr. Girish Mour	Computerized tacrolimus dosing system	Immunosuppression/pharmacokinetics	PK-based computerized tacrolimus dose adjustment	Early post-transplant tacrolimus management	80%–90% within target range; fewer above-goal levels; shorter time to target in high-risk patients	Previously published clinical system	Early deployment stage. Needs further evaluation.
	Tacrolimus dose predictor	Immunosuppression/pharmacogenetics	Predicts stable tacrolimus dose using pharmacogenetic and clinical data	Personalized tacrolimus dosing after kidney transplant	Outperformed traditional regression methods	Internal and external validation cohorts	Predictive model trained on retrospective data. Still requires prospective evaluation.
	Mayo tacrolimus AI decision-support tool	Clinical decision support	Integrates transplant history, medications, labs, recommendations, and clinician feedback	AI-assisted tacrolimus dose recommendation	70–80% accuracy in first testing phase	Tested by 15 clinicians in a simulated environment; not yet clinically deployed	Predictive model trained on retrospective data. Still requires prospective evaluation.
Dr. Joseph Ahn	TabDDPM	Synthetic tabular data	Generates synthetic cohorts while preserving distributions, correlations, and survival structure	Shareable transplant datasets and safer multicentre model development	Low Wasserstein distance; preserved clinical correlations and survival trends	Applied to UNOS liver transplant registry and Mayo liver transplant data	May inherit biases from the original source of data.
	Multi-site synthetic training model	Synthetic data/multicentre generalization	Merges site-specific synthetic cohorts into a single training set	Improves cross-site mortality prediction	AUROC 0.82 vs. 0.79 for single-site model; Florida 0.84 vs. 0.73	Externally tested across Rochester, Arizona, Florida, and MCHS cohorts	May inherit biases from the original source of data.
	In-silico trial/digital twin framework	Virtual trials/counterfactual prediction	Simulates treatment arms and patient-level “what-if” outcomes	Fibrosis screening trials and HCC treatment simulation	Median survival benefit 7.2 vs. 7.5 months; HR 0.57 vs. 0.65 vs. real-world analysis	Parallel comparison with real-world propensity-score-matched data	May inherit biases from the original source of data.
Dr. Walter Kremers	Statistical framework for ML/AI models	Biostatistics/methodology	Frames lasso, ridge, random forest, boosting, and neural networks within loss minimization and likelihood theory	Guides model choice and evaluation in transplant AI	Not model-specific	Conceptual framework; emphasizes cross-validation, calibration, and selective use of complex models	Conceptual/methodologic framework. Not a deployable model.
Dr. Pranav Rajpurkar	Confidence-gated human–AI collaboration model	Human–AI collaboration/radiology	Uses clinician confidence and AI confidence to determine when AI should be shown	Diagnostic workflow design applicable to transplant AI settings	AI outperformed 78% of radiologists by AUROC and 90% by RMSE; 10 percentage-point error reduction when clinician uncertain and AI confident	Randomized study of 227 radiologists; heterogeneity follow-up in 140 radiologists	
	CRAFT-MD	Clinical LLM evaluation	Simulated patient conversations to evaluate history-taking and diagnostic reasoning	AI-first intake and documentation workflows	Large gap between benchmark-style testing and conversational readiness	Early real-world style evaluation framework	
	ReXplain	Patient education/radiology communication	Generates patient-facing video explanations from radiology findings	Doctor-first explanatory workflow	Qualitative physician support; transcript did not provide a single summary metric	Early physician evaluation study	
Dr. Soumita Ghosh	Multimodal liver graft injury biomarker	cfDNA methylation/multimodal ML	Integrates 201 Boruta-selected methylated sites with 8 clinical variables in a neural network	Non-invasive diagnosis of TCMR, MASH post-transplant, and controls	Mean weighted AUROC 0.911	Two-centre cohort; 125 patients	Retrospective proof-of-concept study. Not yet prospectively studied.
Dr. David Pellow	LT-CVD	Post-transplant cardiovascular risk	Predicts 10-year major adverse cardiovascular event risk and displays recommendations in Epic	Long-term cardiovascular risk stratification after liver transplant	Better than general-population scores; 57% action rate during deployment vs. 32% pre-deployment	7-week pilot in liver transplant clinic; 65 patients; multicentre validation ongoing	Early pilot study. Needs multicentre validation.
Serena Hacker	COPD/IPF EVLP digital twin	Lung digital twins/preclinical modelling	Uses physics-based perturbation plus GRU forecasting to simulate diseased *ex vivo* lungs	Preclinical drug testing for end-stage lung disease relevant to transplantation	Low MAE and MAPE across parameters and disease phenotypes	Tested on synthetic COPD and IPF EVLP cases derived from historical EVLP data	Preclinical synthetic-lung model. Trained on simulated datal not yet tested on real diseased lungs.
Deween Piyasena	EVLP donor-function predictor	Donor lung physiology/post-transplant lung function	Ensemble regression using EVLP physiology and donor demographics to predict post-transplant FEV₁ and FVC	Pre-transplant estimation of post-transplant allograft function	FEV₁ MAE 0.35 L and 15% error; FVC MAE 0.42 L and 16% error	71 clinical EVLP cases with >1,000 breath-by-breath physiological profiles	Retrospective model. Requires prospective evaluation.

## 2 Methods

This article is a narrative symposium report based on presentations and panel discussions given at the third annual Transplant AI Symposium in Toronto, Ontario. The authors (PM and DR) reviewed symposium recordings, slideshows, and presentation materials to extract the main themes, descriptions of the ML tools used, and the reported outcomes. Quantitative values were checked against the original symposium materials and whenever possible, verified using the corresponding peer-reviewed publications. All speakers approved of their summarized presentations.

## 3 Symposium highlights

### 3.1 AI algorithms for healthcare decision-making and organ matching

Dr. Tuomas Sandholm, co-director of CMU AI and Professor of Computer Science at Carnegie Mellon University, presented a keynote talk on the potential of AI driven organ matching algorithms for kidney exchange. He described the current growing imbalance between demand and supply for kidney transplants and proposed a kidney exchange program as a solution (1), in which incompatible donor-recipient pairs can swap kidneys with other compatible pairs, thus enabling successful transplant. He noted the computational and logistical difficulty of optimizing matches between a large pool of patients, especially with considerations to compatibility. Common algorithms used to aid this process can be categorized as greedy, weighted maximum, and CPLEX. Greedy algorithms are fast; however, suboptimal for complex problems. Weighted maximum algorithms function better than greedy; however, require additional organization for larger cycle caps. CPLEX can identify optimal solutions for complex tasks yet are computationally expensive and do not scale. Next, Dr. Sandholm discussed his novel Branch-and-Price algorithm which optimally solves complex and scalable kidney exchange matching problems, becoming a vital core of several US kidney exchange program (2).

Dr. Sandholm highlighted donor chains as a major innovation to the exchange program that has drastically improved the rate of successful matches. Donor chains are characterized by altruistic donors donating without any specific recipients predetermined, thereby allowing for proceeding matches to happen over a period of time non-simultaneously (3). Donor chains are one of the current global gold standards for kidney exchange. His current work contributes to further optimizing the chain via the incorporation of compatibility scores, transplant success probabilities, and focus on expected outcomes. In unpublished stimulation results presented at the symposium, this approach demonstrated approximately a two-fold increase in the number of successful matches.

Finally, Dr. Sandholm addressed some considerations and challenges associated with transplant optimization including fairness, policy making and ethics. He noted that decision-making polices, such as prioritization of highly sensitized patient populations or patients, can drastically affect outcomes. He concluded by stating the role of humans in deciding the ethical values and goals of the program and using AI to optimally achieve the goal. In addition, he suggested further research avenues for better dynamic matching algorithms and strategies to increase coordination between transplant centers, thereby maximizing efficiency and fairness.

### 3.2 CMV risk stratification post-transplantation

Dr. Aman Sidhu, medical director of the Lung Transplant Program at Toronto, and Yingjie Sun, a machine-learning analyst at University Health Network (UHN), discussed the challenge of cytomegalovirus (CMV) within lung-transplant recipients. CMV, normally a common virus that creates a primary infection in 50% of the world's population, can be reactivated or transmitted through donor organs (4, 5). Even asymptomatic infection can trigger immune dysregulation, leading to downstream consequences of acute rejection and lung allograft dysfunction. There are two current strategies for CMV protection: universal prophylaxis and pre-emptive prevention. Prevention relies on universal prophylaxis with antivirals because pre-emptive therapy requires frequent monitoring and is not well characterized within lung transplant recipients (6). However, prolonged prophylaxis has its own risks. In one cohort study, 95% of discontinuations were due to adverse effects such as neutropenia and renal impairment (7).

To examine whether matching donor and recipient CMV serostatus could mitigate transmission, Sun created a Monte-Carlo simulator of the lung transplant waitlist. The model incorporates patient and donor characteristics, arrival time, patient priority scores, and proposes the addition of CMV matching, with exceptions for the sickest candidates. Simulation results demonstrate that the overall transplant rate (92.1%) matches the real-world transplant rate (92.2%) and that CMV matching has little effect on median time-to-transplant or mortality however, modestly increases the transplant opportunities for the O-blood group and small-stature patients. Given the existing disparities of lower transplant rates for blood group O and short-stature patients (8, 9), Dr. Sidhu posits that CMV matching may be considered as a potential solution to avoid exacerbating inequities. She concludes that multifaceted approach is required, combining new organ allocation policies, extended prophylaxis with new drugs, and improved surveillance post-transplant to reduce CMV-related mortality and morbidity.

### 3.3 Development and deployment of multimodal AI tools in liver transplantation

Dr. Mamatha Bhat, Hepatologist, Assistant Professor of Medicine at the University of Toronto, and co-lead of the Transplant AI Initiative at University Health Network (UHN), presented an overview of AI tool integration in liver transplantation. She opened by noting the inconsistencies and subjectivity present in complex liver transplant candidacy discussions. Hidden biases, differing workflow and clinical practices between transplant centers, and various human confounding factors influencing these important decisions. AI agents may be a potential approach in reducing these biases and optimizing greatest objectivity in the transplant candidacy discussion (10, 11). To address this, her team, composed of Dr. Ghazal Azarfar, computer research associate, and Dr. Bima Hasjim, surgical resident, developed a multi-agent AI transplant selection committee developed to mimic the multidisciplinary nature of a transplant committee. Each AI agent assumed a different role, ranging from transplant hepatologist to cardiologist to social worker. The models together reviewed over 8,000 cases and successfully identified patients who would derive an one-year post-transplant benefit with 92% accuracy (11). Cosine similarity analysis revealed each agent focused on role-specific factors. For example, the cardiologist agent focused on cardiac history. This approach aims to reduce biases and increase efficiency of the transplant candidacy process, and silent trials are currently occurring at various international transplant centers.

Dr. Bhat then addressed current inequities in the Model for End-Stage Liver Disease (MELD)-based liver transplant allocation system, especially emphasizing female patients and patients with cholestatic liver diseases as being disadvantaged and scoring lower for the same severity of illness (12, 13). Her team developed DynaMELD, a deep-learning dynamic model of end-stage liver disease, which incorporates both clinical and laboratory variables. The model was trained with over 53,000 patients listed for transplant in the United States from 2016 and deployed as part of a silent trail with 382 weight-listed patients at UHN from June 2022 to December 2024. It was developed to address allocation disparities and showed improved discrimination; Whether this translates into improved equity requires further prospective validation (14). Moreover, DynaMELD achieved 90-day concordance 0.5% higher than MELD 3.0. (*p* < 0.001) (14). It is important to note that this improvement should be interpreted as a discrimination result and not as evidence of improved equity. Furthermore, DynaMELD results are currently at preprint stage and have not yet been peer-reviewed.

In the post-transplant setting, Dr. Bhat's team initially built a transformer-based model to predict the long-term mortality from cardiovascular events, infection, graft failures, and cancer. Deployment of the model proved challenging and was associated with hallucinatory predictions. Therefore, they refined the tool to a cardiovascular risk prediction model, which was implemented into the EPIC electronic medical record dashboard. Over a four months pilot study, clinical management changed in over 50% of the patients flagged as high risk by the module (15). For graft monitoring, they developed Weighted Long Short-Term Memory Networks (LSTM) which uses longitudinal clinical and laboratory values to diagnose post liver transplant graft fibrosis. Their model consistently outperformed other models at detecting stage two or greater fibrosis with an area under the curve (AUC) of 0.798 (16). However, when compared to transient elastography, the model did not significantly outperform at detecting fibrosis (16). Building upon this, they developed GraftIQ, a multiclass artificial neural network that incorporated clinical input. The model demonstrated high accuracy with an AUC of 0.866 over all six classes of diagnostic liver graft injury (17). The hybrid GraftIQ model, which incorporated clinician oversight, achieved an overall AUC of 0.902. The inclusion of clinical-derived rules improved the model's performance further (17).

Finally, Dr. Bhat highlighted “omics integration, a field of biology focused on the study of biological molecules. Biological processes in post-transplant metabolic-associated steatohepatitis (MASH) and T-cell mediated rejections have very specific methylation patterns (18). As such, her team combined circulating DNA methylation profiles with clinical variables to train an artificial neural network to achieve an AUC of 0.911 in the diagnosis of MASH and rejection. SHapley Additive exPlanations (SHAP) analysis provided further insight into relevant unique regions of methylation, thereby, suggesting the potential role of AI in disease mechanisms as well as diagnosis. Dr. Bhat concluded by emphasizing the need for further clinical trials to evaluate these tools and better understand their impact on patient health outcomes.

### 3.4 Predicting immunosuppression needs in solid organ transplantation

Dr. Girish Mour, a consultant nephrologist, transplant specialist, and Assistant Professor of Medicine at the Mayo Clinic College of Medicine and Science provided an overview of the current landscape in immunosuppression management and identified limitations in the current model of care. Furthermore, he explored the role of AI in immunosuppression need, prediction, and management. Even with decades of progress, there remains significant risk of post-transplant rejection, one of the major concerns following organ transplantation. The risk is especially higher for younger patients and can lead to allograft failure and life-years lost (19, 20). Dr. Mour described the current post transplant immunosuppression management workflow to be heavily taxing, with paper communications and inconsistencies noted. Most transplant centres use manual processes to track tacrolimus and other laboratory values. These factors contribute to delayed response times, inconsistent clinical practices, and hidden biases that affect patient health outcomes.

To address these limitation, Dr. Mour suggested AI tools to increase objectivity and responsiveness of post-transplant immunosuppression management. Some potential integrations include automated lab data review, generating real-time alerts, personalizing immunosuppression management from the patient's electronic medical records, and patient oriented chatbots for education. Størset et al. developed a computerized dosing system which utilizes pharmacokinetic values to achieve ideal tacrolimus levels in 80%−90% of the patients (21). Furthermore, a significant reduction in hyperglycemia was also noted after an eight-week period (21). Dr. Mour suggested that overexposure to tacrolimus may be associated with metabolic complications such as hyperglycemia. Tang et al. compared nine ML pharmacogenetic algorithms for predicting tacrolimus stable doses on renal transplant and noted that they outperformed traditional regression methods (22). Choshi et al.'s study found that the long short-term memory (LSTM) model was able to accurately predict trough levels of tacrolimus in a variety of clinical situations (23). In addition, he emphasized the importance of human supervision of AI due to its susceptibility to bias.

Finally, Dr. Mour discusses the development of Mayo Clinic's enterprise-level AI tool for tacrolimus dosing, which was trained on laboratory data from over 2,800 renal transplant patients. The tool supports clinicians by providing them with AI-generated dose recommendations. Current model accuracy is limited, ranging between 70% and 80%, highlighting the need for further refinement and optimization. Moreover, he highlights limited data availability during the early post-transplant period, generalizability, and potential bias as limitations to AI integration in context of immunosuppression management.

### 3.5 Synthetic data and digital twins in transplant AI

Dr. Joseph Ahn, Assistant Professor of Medicine and Transplant Hepatologist at Mayo Clinic, in Rochester discussed the emerging use of synthetic data in AI research. While AI applications have seen a rapid rise in progress and research, clinical deployment remains limited secondary to privacy regulations and dataset scarcity, often leading to single-center models that fail to generalize in the real-world. Synthetic data carves a promising solution in this landscape, defined as data that is artificially generated through statistical modelling or computer simulation which allows the preservation of patterns and exclusion of confidential patient information (24). Dr. Ahn explained that generative models, such as generative adversarial networks (GANs), variational autoencoders, and diffusion models can learn patterns in real datasets and create synthetic records with highly realistic medical images and reproduction of clinical trends (25). These approaches, he suggests, could support collaborative research and generalizable AI model training by facilitating safe data sharing. Synthetic datasets can lead to *digital twins*, the concept of virtual replications of individual patients. A digital twin could provide synthetic control arms of clinical trials and optimize treatment decisions (24). Regulatory bodies have recently acknowledged the promise of digital twins. The FDA's guidance on digital health encourages in-silico modeling and simulation in medical device evaluation, for example (24).

Dr. Ahn subsequently presented case studies to illustrate the use of these concepts. With the United Network for Organ Sharing (UNOS) liver-transplant registry, his team trained a TabDDPM diffusion model to generate a synthetic transplant cohort that mimed the distribution of real patient variables, trends, and patterns. In a proof-of-concept multi-institutional study, they created synthetic training sets for four independent alcohol-associated hepatitis cohorts and merged them to train a 30-day mortality prediction model. He found that the synthetic-trained model achieved an AUROC of 0.82 when trained across external sites than a model trained on one site (AUROC 0.79). Synthetic data can also be leveraged for in-silico trials. A synthetic cohort of 50,000 patients was used to compare fibrosis screening strategies such as Steatosis-Associated Fibrosis Estimator (SAFE) vs. Fibrosis-4 (FIB4), which replicated real-world findings that SAFE detects more cases while requiring fewer tests. Another digital twin study simulated treatment responses to immunotherapy alone vs. immunotherapy plus trans-arterial radioembolization for patients with advanced hepatocellular carcinoma. The synthetic cohorts produced survival curves and hazard ratios quite similarly to those observed in real-world analyses. Dr. Ahn further described how for image generation, models can perform “N-painting”, which can reconstruct missing parts of an image. Despite these promises, Dr. Ahn cautioned that synthetic data could inherit the biases of the real-world datasets; synthetic data generation models can only learn what the original data contains, which means confounding variables, missing items, or biases can be carried forward (24). High quality, well-curated datasets with rigorous validation is required before deploying synthetic data or digital twins in clinical decision-making (24). He concluded that synthetic data and digital twins are meant to complement but not completely replace prospective randomized studies.

### 3.6 Statistical foundations of AI/ML models

Dr. Walter Kremers, lead Biostatistician at Mayo Clinic's Division of Biomedical Statistics and Informatics and Associate Professor of Quantitative Health Sciences, presented on statistical foundations behind neural network and ML models. In his keynote, he described the statistical foundations behind AI models, traditionally seen as “black box” models. He explained that machine learning models can be differentiated from traditional statistical models by their capacity to handle non-linear, high-dimensional data. He posited that modern ML models are essentially advanced developments of established statistical techniques, specifically referencing LASSO and ridge regression from the penalized model framework. Likewise, both random forest and gradient boosting models attempt minimize loss function like square error or cross entropy. Finaly, he discussed how researchers should choose between classical statistical and AI models. He noted that deep neural networks underperform for small and tabular datasets, where regression and gradient-boosting models are better suited. Furthermore, he highlighted the importance for clinicians and researchers to evaluate predictive models based on cost and gain. The Scientific Registry of Transplant Recipients (SRTR) continue to use LASSO models for survival prediction since elaborate models provide marginal gain for their associated cost. He concluded by emphasizing that the choice of modelling should be based on the research question, the size and quality of the data, and the need for interpretability.

### 3.7 Human-AI collaboration across medical domains

Dr. Pranav Rajpurkar, Associate Professor of Biomedical Informatics at Harvard Medical School, leads a lab developing multimodal AI methods for biomedical decision-making. He opened the keynote by centering the question on whether AI and doctors will achieve better results in tandem vs. either alone. In a randomized study of 227 radiologists interpreting chest radiographs, Dr. Rajpurkar's team found that an AI model outperformed 78% of the radiologists by AUROC and 90% of radiologists using the root-mean-squared error (26), suggesting that AI's diagnostic accuracy sits within the performance range of human experts; yet when radiologists were given AI assistance, there was no average improvement in diagnostic accuracy. The reason behind this was found to be automation neglect, the concept where physicians were weighing the AI outputs as less than what they should have been. Optimal collaboration was seen in situations where clinicians were uncertain and AI was confident; conversely, uncertain AI recommendations harmed performance. Dr. Rajpurkar therefore proposed a gating model in which AI reads confident cases, clinicians manage the remainder, and AI assistance is only shown when clinicians are unsure.

However, Dr. Rajpurkar emphasized that universal AI deployment could harm certain physicians while helping others, thus masking “winners and losers”. A follow-up study showed that substantial heterogeneity in clinicians' responses to AI and factors commonly thought to further human-AI collaboration such as years of experience, subspeciality, or prior AI use did not predict who benefits from AI. Baseline skill remains the key determinant; strong readers remain strong and weak readers remain weak. He further cautioned that AI could result in a de-skilling effect for clinicians using AI as a part of their workflow, as seen in a colonoscopy study where adenoma detection rates declined after AI usage (27).

Despite these caveats to human-AI collaboration, Dr. Rajpurkar highlighted the major successes. The Swedish MASAI trial of over 105,000 women found that with AI-supported mammography, there was a 29% increase rate of cancer detection and a 44% reduction in radiologist workload without increased false-positive rates (28). Other human-AI partnership studies show that routing only the lowest-confidence cases can boost accuracy by up to 20% (29). Building on these results, Dr. Rajpurkar outlined three distinct workflows for human-AI collaboration: an AI-first sequential workflows such as the CRAFT-MD system is where chatbots gather history and AI drafts recommendations before clinician review (30); the doctor-first sequential workflow such as ReXplain which depends on the physician to form the primary interpretation and the AI for enhancement; and third, case allocation, where certain subsets of cases are read by AI and the others by clinicians (31). He concluded that generalist medical AI models, capable of performing multiple tasks across modalities (32), and tools like NotifAI-OS for automated CT-based opportunistic screening are emerging (33); however, success will depend on robust gating mechanisms, evaluation, and awareness that AI adoption can both help and harm clinicians depending on the method of usage.

### 3.8 A multimodal machine learning biomarker for non-invasive diagnosis of liver graft injury

Dr. Soumita Ghosh, postdoctoral fellow at the Bhat Liver Lab, began her presentation by highlighting metabolic dysfunction-associated steatohepatitis (MASH) and T-cell mediated rejection (TCMR) as being primary contributors to graft injury post liver transplant (34, 35). Both diseases can remain a problem even at the one-year post transplant mark. Both result in abnormal liver biochemistry. However, it is vital to differentiate between the two as management varies drastically. Highlighting that biopsy is currently the standard for diagnosing liver injury causes, Dr. Ghosh emphasized the importance of developing non-invasive biomarkers. Her team conducted a two-center study with 125 patients and made use of Boruta feature selection to select 201 informative methylated sites from cell-free DNA in combination with eight clinical variables. These factors were incorporated in several classification models. The combined neural network model achieved the best performance with a mean AUC of 0.911. SHAP analysis revealed methylated sites along circulating DNA as the important features in the classification of graft pathologies. She concluded by highlighting ML biomarkers as a promising avenue for monitoring graft injury.

### 3.9 Long-term cardiovascular risk stratification after liver transplant

Dr. David Pellow, a postdoctoral fellow at the University of Toronto, presented on developing long term cardiovascular risk stratification tools for post-liver transplant patients. Their primary goal was to develop a novel survival model for 10-year risk of major adverse cardiovascular event (MACE) in post-transplant population. He mentioned that prior to this model, clinicians depended on scores such as the Atherosclerotic Cardiovascular Disease (ASCVD), which were derived from the general population. Furthermore, he stressed the need for scores and models to be calibrated to the post-liver transplant population for greater accuracy. Dr. Pellow and his team developed the liver transplant-cardiovascular disease (LT-CVD) model, a MACE model trained on the post-liver transplant population. It outperformed traditional scores and showed good calibration amongst low-, medium-, and high-risk categories. The model was integrated with the EPIC electronic medical record dashboard to provide clinicians with cardiovascular risk, feature breakdown of factors contributing to risk, and management recommendations. Following a seven-week trial deployment at UHN with 65 patients, the team noted risk management actions were taken in 57% of the cases compared to 32% prior to the model deployment. They addressed the small sample size as a limitation and that multi-centre validation of the tool is currently ongoing. This project anecdotally suggests that ML-driven methods may encourage proactive cardiovascular care in liver transplant patients.

### 3.10 Digital twin model of lung disease

Serena Hacker, a ML researcher and engineer at University Health Network discussed the development of novel preclinical models for lung disease using ML and physics-based models. Currently, lung transplant is the only life-saving treatment for end-stage obstructive and interstitial lung disease. Hacker noted that current cell-based and animal models poorly replicate human lung pathology, thereby making it challenging to develop therapies to slow or reverse disease progression. To address this, they developed a digital twin of ex-vivo human lungs using historical perfusion data, which mimics the behaviour of heathy human lungs on ex-vivo lung perfusion (EVLP). Digital twins are virtual simulations of real systems (36). She then describes that currently, they are developing chronic obstructive pulmonary disease (COPD) and idiopathic pulmonary fibrosis (IPF) disease-specific digital twins using synthetic EVLP and physics-based models. they could successfully simulate the high peak pressures and rapid inspiration associated with idiopathic pulmonary fibrosis (IPF), as well as the resistances characteristic of chronic bronchitis. Synthetic EVLP data was used to test gated-recurrent unit-based (GRU) neural network digital twins. The model performed well with low mean absolute error across the parameters for the different lung disease phenotypes. The GRU model demonstrated promise for accurately forecasting the dynamic physiology of human lungs with various disease phenotypes.

### 3.11 Predicting post-transplant FEV₁ and FVC testing using donor lung function

Deween Piyasena, a master's student working with Dr. Andrew Sage presented on predicting post-lung transplant function using ML and donor lung data collected during EVLP. He noted forced expiratory volume in one second (FEV1) and forced vital capacity (FVC) as measurements essential in predicting post-transplant lung allograft function as well as long-term patient survival (37). Previous studies have attempted to predict post-transplant FEV1 using donor demographic information. However, Piyasena noted that this approach had an error percentage between 25% and 30% due to lack of insight on the donor lung function. To isolate the contributing features of the donor lung in post-transplant FEV1 and FVC, ML was integrated with EVLP data and donor demographic data. The study cohort contained 71 clinical EVLP cases with over 1,000 breath-by-breath physiological profiles. Regression-based models were used to predict FEV1 and FVC at multiple time points up to one-year post transplant. With this approach, they were able to predict post-transplant FEV1 in pre transplant contexts with a mean absolute error percentage of 15%, drastically lower than the only donor-demographic based approach. SHAP analysis revealed elastin derived from the EVLP ventilator traces and donor lung predicated capacity. He concluded by highlighting future avenues in incorporating detailed recipient factors to add a personalized aspect to predictive algorithms.

### 3.12 Panel discussions

Dr. John Halamka, Dr. Brad Wouters, Mary Vyas, and Dr. Shaf Keshavjee led the first panel discussion on building multi-centre AI collaboration. They emphasized the need for large multimodal datasets to ensure that models are fair, effective, and safe. Multi-centre collaborations address this by increasing sample sizes and validation across heterogenous populations and practices; thereby, increasing generalizability, statistical power, and mitigating model biases. Moreover, they highlighted data heterogeneity, privacy, interoperability and standardization as some challenges to the field. These can be addressed through the use of federated learning instead of raw data and common data models with standardized preprocessing and feature definitions.

Dr. Michael Brudno, Maneesh Goyal, Dr. Edson Amaro Jr, Dr. Byron Yount, and Farhana Alarakhiya began the second panel discussion by highlighting barriers to data sharing between institutions. They emphasized cultural limitations, lack of trust and misaligned incentives between institutions as more significant challenges compared to technical barriers. Moreover, they noted that the involvement of legal teams later in the process leads to friction. They stated that addressing these barriers require early alignment of incentives and priorities between partner institutions, with an emphasis on sharing and trust. Furthermore, early involvement of legal teams and the push towards the adoption of common data models and practices can greatly contribute to designing affordable and sustainable systems-level integration of these models.

## 4 Conclusion

This symposium illustrated that transplant AI is moving from proof-of-concept work toward selective clinical use. Speakers highlighted both the utilities of transplant AI (*e.g.,* organ matching, CMV risk reduction, candidacy review, graft injury detection, and risk estimation) as well as the limitations (*e.g.,* poor interoperability, hidden bias, overfitting, uncertain generalizability, and the gap between model development and deployment). Better model performance alone does not guarantee better care; workflow design, calibration, and clinical oversight remain central. Work on synthetic data and digital twins suggested a practical way to address privacy and data scarcity yet also reinforced that these methods inherit the limitations of the original dataset and therefore cannot replace validation.

A major cross-cutting theme throughout the symposium was the distinction between technical performance and clinical readiness. Several tools demonstrated strong retrospective performance metrics; however, many were dependent on single-centre datasets, retrospective validation, or preprint-level evidence. Some tools have already demonstrated meaningful integration into practice, such as the branch-and-price kidney exchange algorithm and the LT-CVD cardiovascular risk tool, which was incorporated into an electronic medical record after a pilot trial. Other models, including GraftIQ and cfDNA methylation biomarkers have shown promising external validation results and performance. In contrast, DynaMELD and the multi-agent transplant selection committee remain in earlier stages of testing, namely, silent trial or preprint level publication. Synthetic data frameworks and digital twin models remain in simulation-based testing. As discussed in presentations surrounding DynaMELD and AI-assisted pathology platforms such as GraftIQ, improved discrimination metrics do not always translate into improved patient outcomes. Future work must therefore focus on silent trials, external validation, and prospective evaluation.

The symposium also raised important questions on clinician interaction with AI systems. Rajpurkar et al. highlighted that AI assistance does not always uniformly improve clinician performance and instead creates heterogenous responses across physicians, producing “winners and losers” depending on their baseline skillsets. These findings challenge the assumption that universal AI deployment will necessarily improve care. Moreover, evidence suggesting clinician de-skilling after prolonged AI exposure raises concerns of overreliance on AI systems. These findings reinforce the need for personalized implementation strategies, targeted training, and careful evaluation of how AI systems can impact clinical decision-making.

Taken together, the symposium suggests that the next phase of transplant AI will depend less on novelty alone and more on whether models are clinically relevant, externally validated, and implemented in a clinically meaningful manner. Continued progress in the field will require advances in model performance and rigorous prospective evaluation, with attention to the ethical and clinical impacts of AI-assisted medicine.

### Key messages

- A small number of transplant AI tools have moved beyond internal validation and into early stages of implementation, including kidney exchange optimization and workflow-integrated risk prediction tools.
- Several models show strong performance, including multimodal graft-injury biomarkers, graft fibrosis prediction, and transplant selection tools.
- Synthetic data and digital twin approaches are especially promising but remain early stage.
- Prospective validation, silent trials, and real-world deployment studies are needed to evaluate how AI systems can impact clinical decision-making.

## Data Availability

The original contributions presented in the study are included in the article/Supplementary Material, further inquiries can be directed to the corresponding author.
